# Infestation of Rice Striped Stem Borer (*Chilo suppressalis*) Larvae Induces Emission of Volatile Organic Compounds in Rice and Repels Female Adult Oviposition

**DOI:** 10.3390/ijms25168827

**Published:** 2024-08-13

**Authors:** Chen Shen, Shan Yu, Xinyang Tan, Guanghua Luo, Zhengping Yu, Jiafei Ju, Lei Yang, Yuxuan Huang, Shuai Li, Rui Ji, Chunqing Zhao, Jichao Fang

**Affiliations:** 1 College of Plant Protection, Nanjing Agricultural University, Nanjing 210095, China; 2020202036@stu.njau.edu.cn (C.S.); 15295595233@163.com (S.Y.); 13611510451@163.com (X.T.);; 2 Jiangsu Key Laboratory for Food and Safety-State Key Laboratory Cultivation Base of Ministry of Science and Technology, Institute of Plant Protection, Jiangsu Academy of Agricultural Sciences, Nanjing 210014, Chinalishuai@jaas.ac.cn (S.L.); jirui@jaas.ac.cn (R.J.)

**Keywords:** volatilomics, transcriptome, plant–insect interactions, rice, rice striped stem borer

## Abstract

Plants regulate the biosynthesis and emission of metabolic compounds to manage herbivorous stresses. In this study, as a destructive pest, the pre-infestation of rice striped stem borer (SSB, *Chilo suppressalis*) larvae on rice (*Oryza sativa*) reduced the subsequent SSB female adult oviposition preference. Widely targeted volatilomics and transcriptome sequencing were used to identify released volatile metabolic profiles and differentially expressed genes in SSB-infested and uninfested rice plants. SSB infestation significantly altered the accumulation of 71 volatile organic compounds (VOCs), including 13 terpenoids. A total of 7897 significantly differentially expressed genes were identified, and genes involved in the terpenoid and phenylpropanoid metabolic pathways were highly enriched. Correlation analysis revealed that DEGs in terpenoid metabolism-related pathways were likely involved in the regulation of VOC biosynthesis in SSB-infested rice plants. Furthermore, two terpenoids, (−)-carvone and cedrol, were selected to analyse the behaviour of SSB and predators. Y-tube olfactometer tests demonstrated that both (−)-carvone and cedrol could repel SSB adults at higher concentrations; (−)-carvone could simultaneously attract the natural enemies of SSB, *Cotesia chilonis* and *Trichogramma japonicum*, and cedrol could only attract *T. japonicum* at lower concentrations. These findings provide a better understanding of the response of rice plants to SSB and contribute to the development of new strategies to control herbivorous pests.

## 1. Introduction

Plants often experience abiotic and biotic stresses during their growth, significantly altering their metabolic pathways [1]. To adapt to or resist these stresses, plants accumulate primary and secondary metabolites [2]. These metabolites (e.g., flavonoids, aldehydes, alcohols and esters) play crucial roles in plant growth and development and serve as important defence pathways in plant responses to herbivorous stress [3]. Some of these compounds are in gaseous form and released through leaf stomata, forming volatile signalling molecules that attract natural enemies or warn neighbouring plants [4,5]. Under herbivore attack, plants activate a series of physiological and biochemical reactions that activate plant defence responses and lead to the production of toxic secondary metabolites (e.g., the release of herbivore-induced plant volatiles [HIPVs]) [6], which regulate the production of defensive compounds, and thus confer resistance to herbivores [7]. Furthermore, HIPVs also serve as informational compounds that attract parasitic or predatory natural enemies to indirectly combat damage due to pests [8,9].

When herbivorous insects attack plants, plants release volatile organic compounds (VOCs; e.g., alkanes, alkenes, ketones, aldehydes, alcohols and esters). Plants might attract natural enemies or resist pests [10]. A study found that the damage caused by lepidopteran larvae induced changes in the proportion of green leaf volatiles emitted by plants. This alteration provides specific and targeted information on pests’ natural enemies, enhancing their efficiency in searching for and locating pests [11]. Additionally, the involvement of phytophagous insect elicitors in the production of HIPVs has been reported [12,13]. The compounds isolated from the oral secretions (OSs) of beet armyworm caterpillars can induce seedlings to emit VOCs and attract parasitic wasps. As an elicitor, OSs trigger plant defence and influence insect behaviour via volatiles [12].

Push and pull headspace collection has been used in volatile organic compound (VOC) collection. The accuracy and efficiency of untargeted methods are limited by multiple factors (e.g., establishing a standard library to ensure the comparability of results). The characteristics or polarity of adsorbent materials and eluents can also affect the types and quantities of detected compounds, which may not provide sufficient sensitivity for substances with extremely small amounts or molecular weights [14]. The untargeted method analyses all detectable metabolites in a sample but has poor reproducibility and identification coverage and a low limit of detection [15,16]. The literature has adopted a widely targeted method for plant volatilome studies (WTV) to overcome these limitations that combines solid-phase microextraction GC-MS/MS (SPME) with a modern analysis pattern [17]. The WTV method provides an efficient, sensitive and solvent-free sample pretreatment technique by directly extracting volatile and semi-volatile compounds from the headspace in the solid-phase matrix of a sample [18]. This method significantly improves the detection range and accuracy of volatile plant metabolites [19]. Transcriptomics, used to study gene expression, can reveal changes in plant gene expression under stress conditions [20]. Combining transcriptomics with volatile metabolomics allows for a comprehensive analysis of plant stress response mechanisms and the identification of key metabolic pathways and regulatory genes. This combined analysis method deepens the understanding of the plant metabolic regulatory network and provides important molecular targets for plant stress resistance breeding and molecular improvement [21].

Rice serves as a major food crop for approximately half of the global population [22]. However, its production is compromised by various pests, including the brown planthopper (BPH, *Nilaparvata lugens*), rice leaf folder (RLF, *Cnaphalocrocis medinalis*), and the rice striped stem borer (SSB, *Chilo suppressalis*) [23]. The SSB stands out as an exceptionally destructive pest, posing a severe threat to the food security of rice yields [24]. An infestation by the SSB can lead to devastating losses, resulting in substantial economic repercussions [25]. This study used a HS-SPME-GC-MS (headspace solid-phase microextraction-gas chromatography-mass spectrometry) approach combined with RNA sequencing to examine the interactions between rice and rice striped stem borers (SSB, *Chilo suppressalis*) by identifying VOCs and related genes associated with the direct and indirect defence of rice plants against SSB attack. We compared SSB adult oviposition performance under SSB larvae-infested with that of the control group, analysed the transcriptome and volatile metabolome profiles of rice infested with SSB and studied the key defence-related genes and secondary metabolites involved in SSB adult behaviour. We aimed to identify key target genes involved in the regulation of rice–SSB interactions and provide insights into how VOCs contribute to rice defences against SSB and a scientific basis for the development of crop varieties with stronger stress adaptability.

## 2. Results

### 2.1. Pre-Infestation of Rice by SSB Larvae Reduced Subsequent SSB Female Adults Oviposition

**Figure 1:**
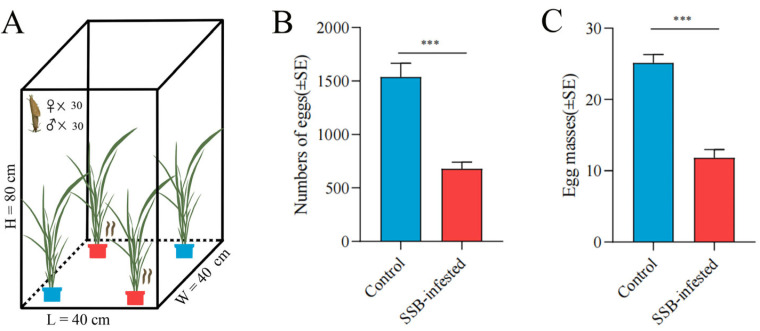
Oviposition preference of SSB in different treated rice plants. (**A**) A scheme of the oviposition experiments; (**B**) Number of eggs laid by SSB female adults in uninfested and SSB pre-infested rice plants; (**C**) Numbers of egg masses by SSB female adults in uninfested and SSB pre-infested rice plants. The experiment was continued for 72 h and repeated 15 times. Statistical significance was calculated using SPSS. Each bar represents the mean ± SE. Data analysed using GLMs with Wald χ2 statistics indicate the overall difference between uninfested rice and SSB pre-infested rice plants. *** *p* < 0.001 indicate significant differences between comparison groups.

When given an oviposition behaviour choice between uninfested rice and SSB pre-infested rice plants, SSB female adults strongly preferred to lay eggs on uninfested control plants than on SSB caterpillar pre-infested plants (Figure 1A). The average number of eggs in each control group was 1538, higher than that in the SSB-pre-infested rice plants (677) (Figure 1B). The average number of egg masses in each control group (25) was higher than that in the SSB-pre-infested rice group (12) (Figure 1C). The average of these 15 replicates showed that the number of eggs and egg masses in the SSB-pre-infested rice was significantly lower than those in the control group. These results imply that SSB female adults prefer to lay eggs on healthy rice plants and that SSB pre-infested plants have a negative regulatory effect on the subsequent oviposition behaviour of SSB female adults.

### 2.2. VOC Analysis of SSB-Infested Rice Plants

**Figure 2:**
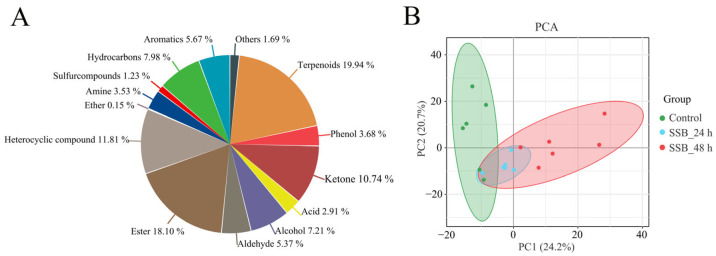
Volatile organic compounds analysis in rice plants. (**A**) Classification and proportion of 650 VOCs detected in rice plants. (**B**) Principal component analysis (PCA) among samples of rice plants by HS-SPME-GC-MS in different groups; the X-axis and Y-axis represent the first and second principal components, respectively.

In total, 650 VOCs were identified in the three rice plant treatments by using HS-SPME-GC-MS (Headspace solid-phase microextraction chromatography-mass spectrometry) (Figures S1 and S2). Detected VOCs were mainly divided into 13 groups: terpenoids, esters, heterocyclic compounds, ketones, hydrocarbons, alcohols, aromatics, aldehydes, phenols, amines, acids, sulphur compounds and ethers. Terpenoids contributed the most (19.94%), followed by esters (18.10%) and heterocyclic compounds (11.81%) (Figure 2A). Principal component analysis showed that the first principal component explained 24.2% of the total variance and separated the samples per the different treatments in each group. The second principal component accounted for 20.7% of the total variance. Samples with three biological replicates were gathered for each group and significantly varied among the groups (Figure 2B).

**Figure 3:**
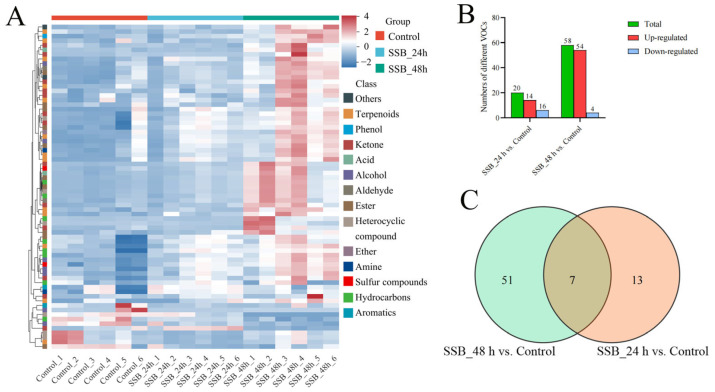
Overall analysis of VOC changes in rice plants’ response to SSB infestation. (**A**) Hierarchical cluster analysis of differentially accumulated VOCs in three treatment groups (SSB_24 h, SSB_48 h, Control); each group contained three biological replicates. (**B**) A histogram with VOCs in SSB-infested rice plants compared with the control group. (**C**) Venn of VOCs in two comparison groups.

Hierarchal clustering of the VOC profiles in the three rice treatment groups was performed (results shown as a heatmap in Figure 3A). The VOCs varied significantly among the rice samples per the variable influence on projection (VIP) of the OSC-partial least squares-discriminant analysis (OPLS-DA) model (Figures S3 and S4). The differentially accumulated VOCs among the three treatment groups were analysed. The amount of differentially accumulated VOCs in the SSB_48 h vs. control comparison group (58) was higher than that in the SSB_24 h vs. control comparison group (20). Among the 20 differentially accumulated VOCs in the SSB_24 h group compared with the control group, 14 were upregulated, and 6 were downregulated. In the SSB_48 h vs. control comparison group, there were 54 upregulated and 4 downregulated VOCs (Figure 3B and Figure S5). These results suggest that the VOC accumulation changed dynamically among the treatment groups of rice plants, and differences increased as treatment time increased. Seven differentially accumulated VOCs were in the SSB_24 h and SSB_48 h groups. Thus, VOCs largely accumulated after 48 h of SSB infestation (Tables S1 and S2). The number of VOCs increased significantly with the SSB-infested time. The differential accumulation of VOCs may be an important direct or indirect defence mechanism of rice plants under SSB damage (Figure 3C, Table S3). Thus, we analysed these significantly upregulated or downregulated VOCs.

**Figure 4:**
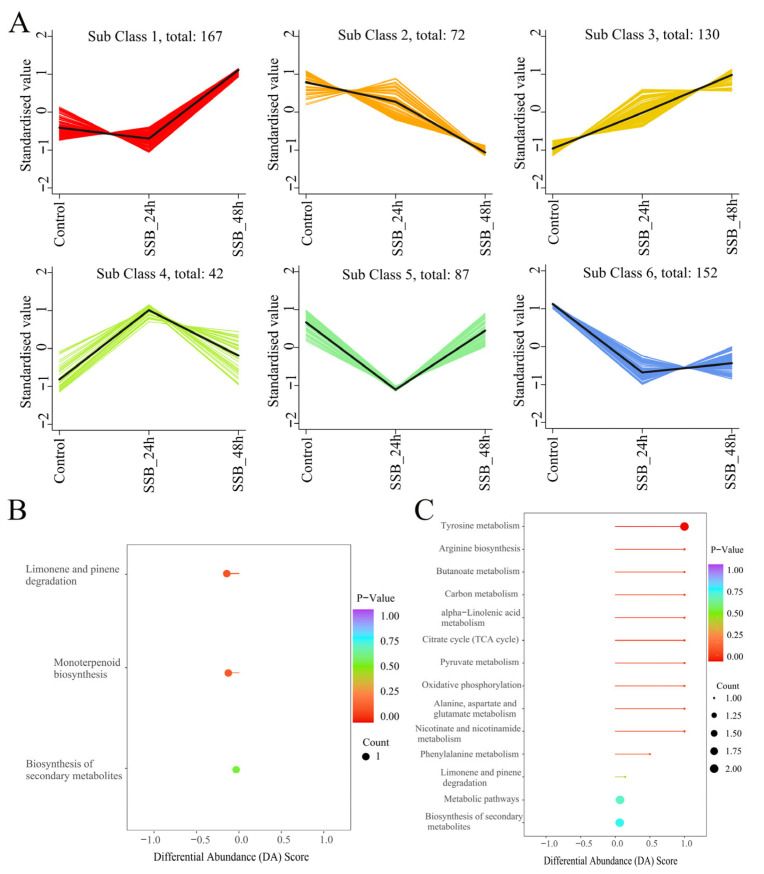
K-means plot and KEGG pathways enrichment of VOCs metabolome in SSB-infested rice compared with the control group. (**A**) VOCs from different samples collected from control, 24 h and 48 h after treatment were used for the K-means plot. KEGG pathways enrichment of VOCs metabolome in SSB_24 h vs. Control (**B**) and SSB_48 h vs. Control comparison groups (**C**).

Next, we investigated the differences in the identified VOCs in response to SSB infestation. Co-expression analysis showed that approximately seven VOCs were significant in each compared group, which were all significantly upregulated in each compared group. The upregulated VOCs included two terpenoids and two esterands. K-means clustering was used to analyse the trend in the relative content of VOCs in the treatment groups, revealing six major trends for all VOCs. Subclasses 1 and 3 had upward trends, and subclasses 2 and 4 had downward trends. Most defence-related secondary metabolites in clusters 1 and 3 included terpenoid substances, which were upregulated in the rice treatment group compared with the control group. Clusters 2, 4 and 6 mainly included esters, alcohols and terpenoids, which were downregulated in these treatment groups. These secondary metabolites showed similar expression patterns in all treatment groups (Figure 4A). Enrichment analysis showed that the Kyoto Encyclopedia of Genes and Genomes (KEGG) pathway was significantly enriched in monoterpenoid biosynthesis, limonene and pine degradation, tyrosine metabolism and alpha-linolenic acid metabolism (Figure 4B,C). These findings indicate that the metabolite profile of VOCs is associated with the response of rice plants to SSB attack, which is probably involved in the induction of direct and indirect defence in rice plants.

### 2.3. Responses of Rice Plants to SSB Infestation at the Transcriptional Level

**Figure 5:**
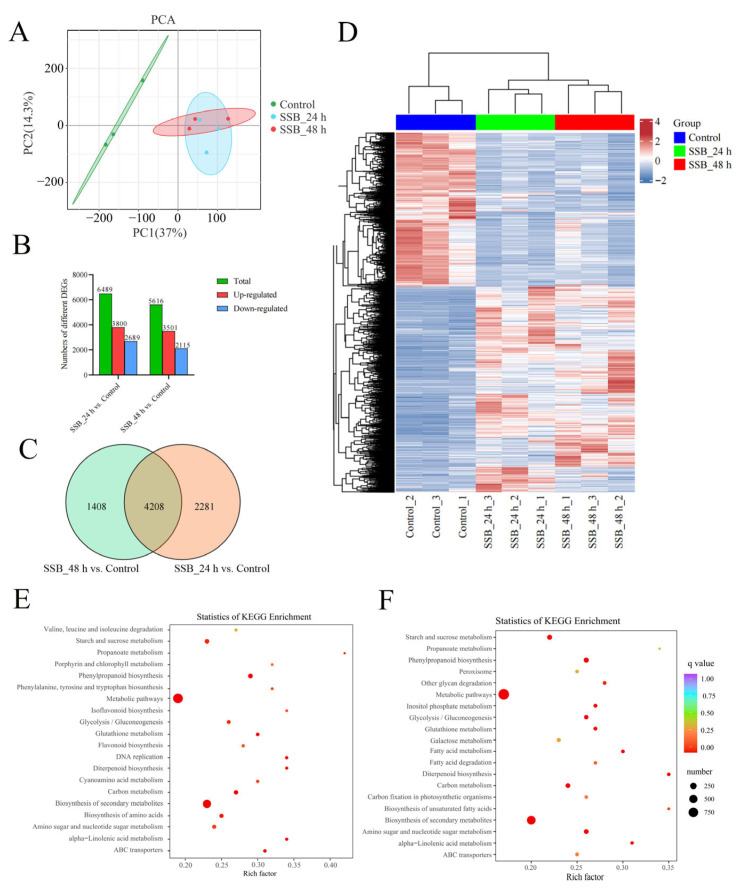
Overall analysis of differentially expressed genes changes in rice plant response to SSB infestation. (**A**) Principal component analysis of each transcriptome sample; X-axis, Y-axis and Z-axis represent the first, second and third principal components, respectively. (**B**) A histogram with DEGs in plants infested by SSB compared with control group. (**C**) Venn of DEGs in two comparison groups. (**D**) Hierarchical cluster analysis of DEGs in the three groups of SSB-infested; each group contained three biological replicates. (**E**,**F**) Bubble plots with KEGG pathways enriched for DEGs in rice infested by SSB at the 24 h and 48 h time-point compared with the control group.

To identify valuable direct and indirect defence-related genes and VOCs in rice adaptation to SSB feeding, we analysed the underlying mechanisms of interactions between SSB and rice at the transcriptional level. The transcriptomes of the SSB-infested and uninfested plants were characterised using Illumina RNA sequencing at three time points: 0, 24 and 48 h. Nine cDNA libraries were observed (Figure S6), resulting in approximately 43–62 million clean reads. The GC content accounted for 50.23–54.98% of these reads. The average number of reads mapped to the rice reference genome was >88%. Unique mapping rates ranged from 73% to 97%. Unique matching reads were used for further analysis. Gene structure analysis revealed that most of the mapped reads (80%) were distributed in exons. In total, 56,637 genes were successfully assembled and annotated (Tables S4 and S5). Principal component analysis showed that differences between groups were greater than differences within groups (Figure 5A). The three groups were assigned to three quadrants of the principal component analysis (PCA) diagram. The comparison of the differentially expressed genes (DEGs) in the groups relative to the control (SSB_24 h vs. Control; SSB_48 h vs. Control) resulted in 6489 and 5616 DEGs with significantly altered expression levels, respectively. More genes were upregulated than downregulated: compared with the control group, 3800 genes were upregulated and 2689 were downregulated in the SSB_24 h group, and 3501 genes were upregulated and 2115 were downregulated in the SSB_24 h group (Figure 5B and Figure S7). A Venn diagram of this dataset indicated that 1408 and 2281 genes were differentially expressed in the SSB_24 h vs. Control and SSB_48 h vs. Control groups, respectively (Figure 5C). Centralised and normalised FPKM (Fragments per kilobase of transcript per million fragments mapped) expression of the differentially expressed genes (DEGs) was extracted to construct a hierarchical cluster analysis (HCA) map (Figure 5D). Kyoto Encyclopedia of Genes and Genomes (KEGG) pathway enrichment analysis of all the DEGs revealed over 30 pathways (Tables S6 and S7), including the biosynthesis of secondary metabolites, phenylpropanoid biosynthesis, metabolic pathways, amino sugar and nucleotide sugar metabolism, diterpenoid biosynthesis, carbon metabolism, glycolysis/gluconeogenesis, glutathione metabolism, inositol phosphate metabolism, alpha-linolenic acid metabolism, and starch and sucrose metabolism, were significantly enriched in all test groups. Fatty acid metabolism, isoflavonoid biosynthesis, flavonoid biosynthesis, benzoxazinoid biosynthesis, flavone, flavonol biosynthesis and biotin metabolism were slightly enriched in each group (Figure 5E,F). The top 20 enriched pathways for the comparison of these groups shown in gene ontology (GO) analysis revealed that the DEGs were mostly involved in biological processes, with significant enrichment in cellular processes, metabolic processes, responses to stimuli, biological regulation and regulation of biological processes (Figure S8) (Tables S8 and S9). These enrichment analyses indicated that SSB infestation strongly influenced various biochemical processes in rice plants, especially in the SSB_24 h samples.

### 2.4. Correlation Analysis between the Transcriptome and Metabolome

**Figure 6:**
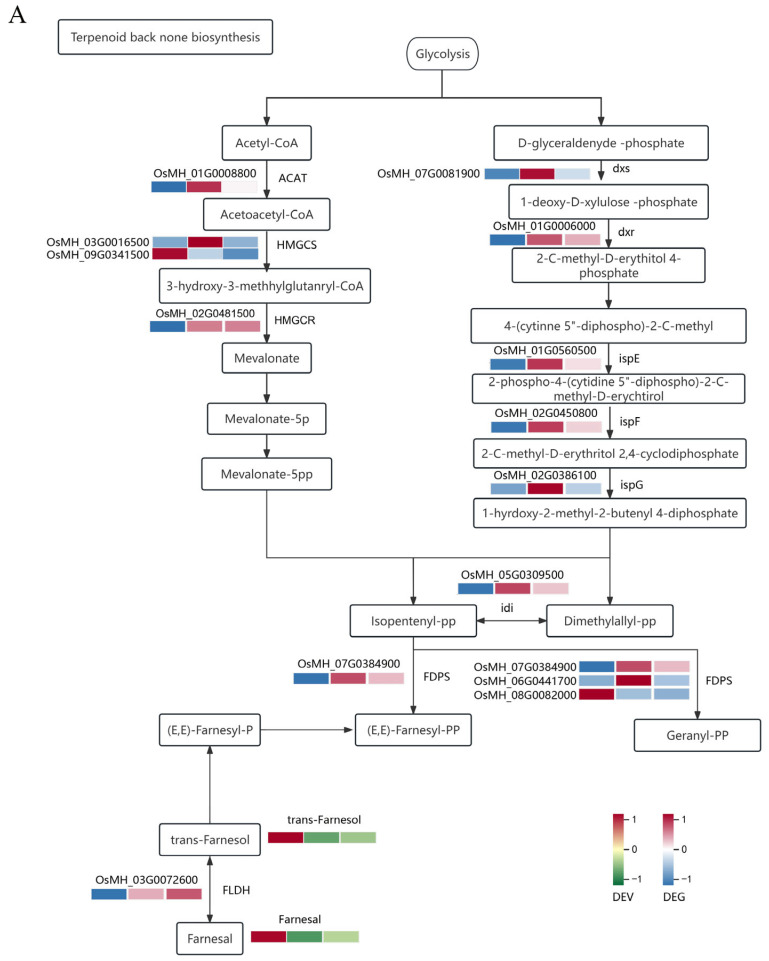

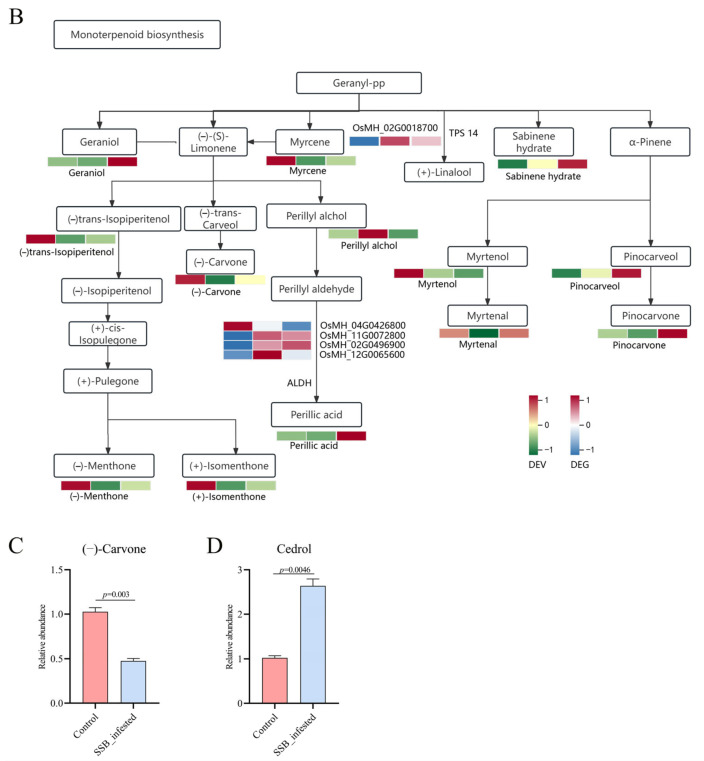
Analysis of terpenoid biosynthesis and differences between the three treatment groups in rice and GC-MS validation. Note: (**A**) Key structural genes and their expression level involved in terpenoid backbone biosynthesis pathway in rice in treatment groups. (**B**) Expression levels of differential metabolites in monoterpenoid biosynthesis pathway. DEV represents differentially expressed volatiles; DEG represents differentially expressed genes; red represents high expression levels; green and blue represent low expression levels in volatiles and transcript, respectively. (**C**) Relative abundance of (−)-carvone compared with SSB_induced to Control group in GC-MS. (The values represent the mean percentages ± SE of the peak area relative to the peak area of the internal standard). (**D**) Relative abundance of cedrol compared with SSB_induced to Control group in GC-MS. (The values represent the mean percentages ± SE of the peak area relative to the peak area of the internal standard).

To analyse the relationship between transcriptional regulation and volatile metabolite accumulation in terpenoid compounds, we performed KEGG pathway enrichment analysis on differentially expressed VOCs and DEGs and a comprehensive analysis of transcripts and VOCs related to terpenoid synthesis pathways. Enriched in the KEGG pathway were monoterpenoid biosynthesis and terpenoid backbone biosynthesis. Specific co-enrichment to trans-farnesol, farnesal, (−)-carvone, fumaric acid, fumaric alcohol, geraniol and myrcene were substances such as (−)-trans isoperitenol, perilly alcohol, (+)-isementhone, (−)-menthone, sabinene hydrate, myrtenol, pinocarveol and pinocarvone. The genes enriched in related pathways included significant DEGs as follows: ACAT, HMGCS, HMGCR, dxs, dxr, ispE, ispF, ispF, idi, FDPS, FLDH, TPS14 and ALDH. The integrated pathway results showed that the differential VOCs and genes involved in terpenoid synthesis had different accumulation expression patterns in the treatment groups, and regardless of the differential treatment, they had a related trend of change as downstream metabolites. In the terpenoid synthesis pathway, volatiles trans-farnesol and farnesol showed lower levels after SSB attack treatment than those of the control group; the significant DEG FLDH showed low expression in the control group, high expression after SSB treatment and the highest expression in the SSB_48 h group (Figure 6A). FLDH might negatively regulate trans-farnesol and farnesol. This regulatory trend was consistent with perillic acid showing an increase in detectable volatile content after 48 h of SSB attack treatment, consistent with the regulatory trend of the differentially expressed ALDH gene (Figure 6B). qRT-PCR experiments were performed on terpenoid metabolite synthesis pathway genes, including dxr, ALDH3, ALDH2, FLDH, FDPS, HMGCR and ACAT, which may be involved in the regulation of terpenoid content (Figure S9). We compared the qRT-PCR results and the transcript abundance (Fold Change) obtained via transcriptome sequencing; the expression levels of the eight genes were consistent with the RNA-Seq expression data, supporting the reliability of the transcriptome sequencing data obtained in this study. Some compounds in the diterpenoid synthesis pathway had similar correlations with genes. Thus, these DEGs might play important roles in the differential accumulation of volatile terpenoid metabolites in rice under different treatments.

### 2.5. GC-MS Validation and Behaviour Response on SSB and Parasitic Wasps to Candidate Vocs

The volatile compounds (−)-carvone and cedrol were collected and identified from the headspace of rice plants by using GC-MS/MS methods and compared with the SSB-infested and Control groups by using the peak area. The (−)-carvone content in the SSB-infested group was lower than that in the control group. Cedrol content in the SSB-infested group was significantly higher than that in the control group. These results are consistent with the trend measured using the GC-MS. Relative abundances are shown in Figure 6C,D.

**Figure 7:**
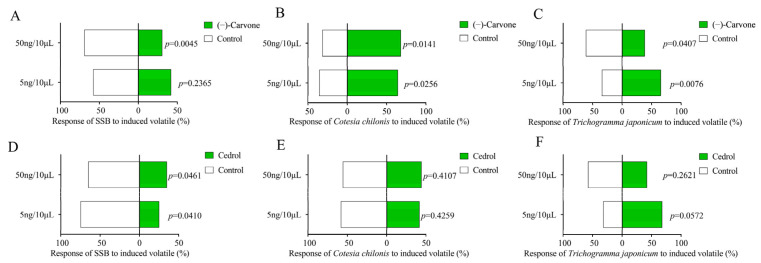
Behaviour response to significant chemical compounds in monoterpenoid biosynthesis pathway to SSB; natural enemy *Cotesia chilonis*, *Trichogramma japonicum*. (**A**–**C**) show preference for SSB, *C. chilonis*, and *T. japonicum* behaviour response to chemical compounds (−)-carvone, respectively. (**D**–**F**) show the preference of SSB, *C. chilonis*, and *T. japonicum* behaviour response to chemical compounds cedrol, respectively.

At low concentrations, SSB female adults showed no behavioural response to (−)-carvone; at high concentrations, they displayed avoidance behaviour towards (−)-carvone (Figure 7A). SSB preferred the control group to avoid (−)-carvone compounds. *Cotesia chilonis* was attracted to low and high concentrations of the compound (Figure 7B). *Trichogramma japonicum* had a strong attraction behaviour at low concentrations and at high concentrations, a tendency to avoid and escape from (−)-carvone (Figure 7C). Another selected VOC was cedrol; at both concentrations, SSB adult females showed significant avoidant behaviour towards the compounds (Figure 7D). For these two parasitic wasps, there were no significant differences in the behavioural response, except for *T. japonicum*, which showed attraction at lower concentrations (Figure 7E,F).

## 3. Discussion

Plants release volatiles during their entire growth cycle that play important roles in physiological activities [18], which are affected by environmental stresses and biological threats [26]. Studies on VOCs have provided compositional and chemical information on rice plants and the formation of metabolic products in the volatile compounds that provide the target. We combined WVT methods with transcriptome analysis to explore the types and specific quantities of volatiles. VOC accumulation and gene expression changes in the treatment groups were observed. Enrichment analysis showed that the VOCs and transcripts had different response pathways. Transcription response was faster than metabolic accumulation, and the rice plant gene response at SSB_24 h was stronger. The VOC enrichment results at SSB_48 h showed the highest metabolism (Tables S1 and S2).

Studies on the volatile metabolites of rice plants have focused on specific classes of insects, and the types and numbers were less than 100 [27]. Even among researchers from the same laboratory, there are differences in the reproducibility of the types and quantities identified in different experiments. However, the overarching differences in the metabolic profiles of SSB attacks remain unclear. The number of identified VOCs using WTV methods was larger than that of the traditional untargeted method (Table S10) [6]. For example, 52 and 145 tomato volatiles were detected using the untargeted and WTV methods, respectively. The number of annotated signals increased from 43 to 132 in rice grains by using the untargeted and WTV methods [17]. This study identified 650 VOCs, including reported chemical compounds (Table S10). The progressiveness and accuracy of the method ensured the reliability of the experiment while enabling more volatile signals to be recognised. The widely targeted method has higher sensitivity, specificity and excellent quantification ability while retaining high metabolome coverage. This difference provides more selectivity for the presentation of experimental results, additional possible targets and new horizons for research and analysis.

This study used more accurate identification of VOCs combined with transcriptomic techniques to identify and quantify the types of VOCs induced by different treatments and to find evidence of their correlation through combined analysis with terpenoid synthesis pathways to provide ideas for the utilisation of chemical ecological control (Figure 6A,B). In this study, two compounds were used for the bioassay. The (−)-carvone was enriched in the terpenoid synthesis pathway (Figure 6B), but no relevant functional studies were conducted. The compound cedrol is in the terpenoid class, and although it was not successfully enriched in the terpene synthesis pathway. Despite reports of it being associated with parasitic wasps of rice planthoppers, no positive behavioural reactions have been observed (Figure S10) [6].

Volatiles released by plants through their metabolic activities during natural growth and under stress play important roles (e.g., signals that can be captured by insects and neighbouring plants). These types of important compounds are beneficial for insect–plant collaboration and long-term evolution, and some may be simultaneously utilised by multiple insects or insects and plants. For example, (E)-β-farnesene, a terpenoid compound, is a secondary metabolic product [28]. This alarm pheromone is recognised by aphids [29] and is released from their abdomen when they encounter attacks from natural enemies or other dangers; it is used to alert aphids and guide their behaviour. (E)-β-farnesene is present in plants, and when rice is persecuted by pests, E-B-F compounds are released and (E)-β-farnesene can damage SSB [30]. We identified the derivatives and isomorphic substances per trans-farnesol (Figure 6A).

These compounds released by plants induced by pests are utilised by insects and natural enemies and are recognised and utilised by neighbouring plants. Tea plants infected with Ectropis oblique caterpillars released indole [31]. When neighbouring tea plants are exposed to indole, the expression of early defective genes involved in calcium (Ca^2+^) signalling, MPK signalling and the production of defence-related secondary metabolites, including jasmonates, is observed. Maize plants use induced volatile (Z)-3-hexenyl acetate and aromatic volatile indole to detect herbivore- and pathogen-attacked neighbours and prime their defences [32]. We identified these volatile chemical compounds, and (Z)-3-hexenyl acetate was significantly upregulated (2.92-fold) in rice subjected to SSB attack (Table S2). Plants attacked by aphids released methyl salicylate. This volatile compound plays an important role in plant defence against herbivorous insects (e.g., aphids) [33]. They participate in plant defences by repelling insects, reducing their adaptability and attracting natural enemies. Plants attacked by aphids release volatile compounds to elicit airborne defence in neighbouring plants. However, aphid-transmitted viruses interact with NAC2, inhibiting the airborne defence response of plants [5].

The functions of various green leaves and herbivore-induced volatiles released by plants in the interactions between insects and plants are complicated. Despite the many compounds emitted by plants, knowledge of compounds with identified functions is limited. Despite research on VOCs and progress in the chemical ecology of insect–plant interactions, many compounds that regulate insect behaviour or induce plant defence functions are still unknown [34,35]. However, upgrading testing technology and continuous systematisation and improvement in analysis levels will increase the number of functional compounds being identified [36,37]. By combining next-generation transcriptome sequencing technology with the molecular basis of references, precise pathway validation and gene targets have been provided that will improve research on the formation and regulation of volatile metabolites. Research on these chemical and ecological foundations will reveal the mechanisms of insect–plant interactions and provide a reference for the practical application of volatile metabolites [38]. It also has the potential for application in developing insect repellents and attractants with better utilisation of push–pull policies and plant sources.

## 4. Materials and Methods

### 4.1. Plant Growth and Insect Colony

The Minghui 63 rice (*Oryza sativa*) used in this study was provided by Biorun Biotechnology. Inc. (Wuhan, China). The rice seeds were first treated with 1% sodium hypochlorite for 15 min, then washed three times with sterile water. The rice seeds were planted into a 600 mL plastic pot filled with sterile soil under controlled conditions of a constant temperature of 28 ± 2 °C, 75% relative humidity and a 16 h: 8 h light/dark cycle. The 50-day rice plants were selected for the test [38]. The rice striped stem borers (SSBs) were collected from laboratory colonies (Rice Pest Control Laboratory, Institute of Plant Protection, Jiangsu Academy of Agricultural Sciences, Nanjing, China) with 30 generations. Insect rearing was conducted in indoor conditions under 28 ± 1 °C, 80% relative humidity, 16 h light/8 h dark, and fed with an artificial diet [39]. The *Cotesia chilonis* pupae were obtained from Yangzhou University, which fed on 10% honey solution and were maintained in glass tubes (diameter 3.5 cm, height 12 cm). The *Trichogramma japonicum* eggs were purchased from Fujian Yanxuan Biological Control Tech. Inc. (Fuzhou, China) ready for use.

### 4.2. Plant Treatment with SSB Caterpillars

The third-instar SSB larvae with the same body sizes were used for the test. Firstly, the SSB larvae were selected from the artificial diet and starving for 12 h, then two SSB larvae were placed on each rice leaf sheath and continued drilling and damaging [6]. The rice samples were divided into three treatment groups, including the uninfested rice group (Control); SSB continued drilling and damaging for 24 h (SSB_24 h) and 48 h (SSB_48 h). For a widely targeted method for plant volatilome study, each treatment included 6 biological replicates, which were named as SSB_24 h_1, SSB_24 h_2, SSB_24 h_3, SSB_24 h_4, SSB_24 h_5, SSB_24 h_6, SSB_48 h_1, SSB_48 h_2, SSB_48 h_3, SSB_48 h_4, SSB_48 h_5, SSB_48 h_6, Control_1, Control_2, Control_3, Control_4, Control_5 and Control_6, respectively. For transcriptomic sequencing, each treatment included 3 biological replicates. The fresh weight of each sample containing five rice plants was not lower than 3 g, which were flash-frozen in liquid nitrogen and stored at −80 °C.

### 4.3. SSB Larvae Bioassay

Bioassay was conducted to test the oviposition performance of SSB adults feeding on multiple pre-treated rice groups [34]. The rice plant pretreatment was divided into two groups. Two active third-instar SSB larvae were placed on each rice plant and fed for 24 h, labelled as SSB-infested. The other group consisted of untreated rice samples as a control. The bioassay cage was made of an 80-mesh nylon net and plastic bottom, with a size of 40 cm × 40 cm × 60 cm. The same pre-treated rice samples were located in opposite positions, and four plants from two different treatments were placed in the same cage (Figure 1A). Subsequently, 30 pairs of male and female SSB adults were released in the cage and kept mating for 24 h. After 72 h, the number of eggs on each plant was counted. The experiment was conducted in an indoor condition at 28 ± 2 °C, with 80 ± 10% relative humidity, and a photoperiod of 16 h light/8 h dark; fifteen times replicates were conducted between SSB-infested plants and uninfested plants.

For VOCs induced SSB behaviour test, the olfactometer for SSB adults consisted of a Y-tube, which has a base tube (20 cm length and 36 mm inner diameter) and two lateral arms (each 20 cm length and 36 mm inner diameter) [13]. The olfactometer for wasps was smaller and had a base tube (7 cm length and 10 mm inner diameter) and two lateral arms (each 7 cm length and 10 mm inner diameter); the angle between the two lateral arms was 75°. Each lateral arm was sequentially connected to a gas flowmeter, a glass odour source chamber, a distilled water humidifier, an activated carbon filter and an air pump (MP-E300NII, Sibata Scientific Technology, Saitama, Japan) [27]. Bioassays were performed under a dim red light supplied with a fluorescent tube for SSB adults. SSB adults were introduced individually at the base of the Y-tube and allowed to move into the olfactometer and choose between two lateral arms. Each of them was connected to an odour source chamber, either holding chemical compounds or hexane. The choice was recorded when an SSB reached at least 2 cm along the lateral arms and stayed at least 30 s. Each test was terminated after 10 min from the introduction of the SSB into the Y-tube. A giving SSB was used only once and discarded after the test. A clean Y-tube was used for each test [40]. A total of 24 insects were tested in each group; all chemical compounds used in this study were obtained from Shanghai Aladdin Biochemical Technology Co., Ltd. (Shanghai, China).

### 4.4. Widely Targeted Rice Volatilome by Headspace Solid-Phase Microextraction-Gas Chromatography-Mass Spectrometry (HS-SPME-GC-MS)

Briefly, rice tissues were harvested and ground to a powder in liquid nitrogen, one gram of the powder was transferred to a 20 mL headspace vial containing a saturated NaCl solution. The vials were sealed using crimp-top caps with TFE-silicone headspace septa (Agilent Technologies, Palo Alto, CA, USA). For SPME analysis, each vial was placed at 100 °C for 5 min, then a 120 µm divinylbenzene/carboxen/polydimethylsiloxane fibre (Agilent Technologies, Palo Alto, CA, USA) was exposed to the headspace of the sample for 15 min at 100 °C. For volatile organic compounds (VOCs) analyses, GC-MS conditions of the VOCs from the fibre coating were carried out in the injection port of the GC apparatus (Model 7890B; Agilent Technologies, Palo Alto, CA, USA) at 250 °C for 5 min in the splitless mode. The identification of VOCs was carried out using an Agilent Model 7890B GC and a 7000D mass spectrometer (Agilent Technologies, Palo Alto, CA, USA) equipped with a 30 m × 0.25 mm × 0.25 μm DB-5MS (5% phenyl-polymethylsiloxane) capillary column. Helium was used as the carrier gas at a linear velocity of 1.2 mL/min. The oven temperature was programmed from 40 °C (3.5 min), increasing at 10 °C/min to 100 °C, at 7 °C/min to 180 °C, at 25 °C/min to 280 °C and then held for 7 min. The quadrupole mass detector, ion source and transfer line temperatures were set at 150, 230 and 280 °C, respectively [17]. MS was used to identify volatile compounds by comparing the mass spectra with the data system library and linear retention index. Three biological replicates of each assay were performed.

### 4.5. RNA Preparation and Construction of Sequencing Library

Plant samples were collected from rice plants of three treatment groups, and total RNA was extracted using the TRIzol reagent (Invitrogen, Thermo Fisher Scientific, Waltham, MA, USA). The cDNA libraries were constructed and sequenced on the Illumina sequencing platform; three biological replicates were sequenced for each treatment. Feature Counts v1.6.2 was used to calculate the gene alignment and the Fragments Per Kilobase of the exon model per million mapped fragments based on the gene length [41]. DESeq2 v1.22.1 was used to analyse the differentially expressed genes (DEGs), and the *p*-value was corrected using the Benjamini & Hochberg method [42,43]. The corrected *p*-value and Log_2_foldchange (Log_2_FC) were used as the threshold for significant difference expression. The enrichment analysis was performed based on the hypergeometric test. For KEGG, the hypergeometric distribution test is performed with the unit of the pathway [44]; for GO, it is performed based on the GO term [45].

### 4.6. Vocs Detection and DEG Enrichment

VIP values were extracted from the OPLS-DA results, which also contained permutation plots and were generated using the R package MetaboAnalystR. The data were log-transformed (Log2) and mean centred before OPLS-DA [46]. To avoid overfitting, a permutation test (200 permutations) was performed. All data for each treatment group were statistically analysed using Student’s *t*-test (*p* < 0.05) with GraphPad Prism 8.0 (Version 8.0.2, GraphPad Software, La Jolla, CA, USA), Excel 2013 (Version 15.0, Microsoft Corporation, Redmond, WA, USA) and SPSS Statistics (Version 20.0, IBM Corporation, Armonk, NY, USA). Differentially expressed VOCs were defined as metabolites with Log_2_FC ≥ 1 and VIP ≥ 1. Differentially expressed genes were defined as unigenes with FDR < 0.05 and Log_2_FC ≥ 1; *p* values < 0.05 were considered to be significant when identifying enriched GO terms and enriched KEGG pathways.

### 4.7. Gas Chromatography-Mass Spectrometry (GC-MS)

Volatiles were collected in an artificial climate chamber at a temperature of 28 ± 2 °C and humidity of 75 ± 10% for 4 h [6,47] to analyse the chemical composition of VOCs in rice samples. A total of 10 rice seedlings were placed in a closed resin box (80 × 80 × 100 cm) and served as a source of odour collection. The bottom connection port is discharged through a tube filled with 30 mg Super Q adsorbent (80/100 mesh, ANPEL Laboratory Technologies Shanghai Inc., Shanghai, China). The flow rate controlled by the flow meter is 500 mL/min. HPLC-grade hexane was used to dissolve and elute the substances collected in the adsorbent compounds and stored in a 2 mL chromatography bottle (Agilent Technologies, Palo Alto, CA, USA), kept at 4 °C away from light until detection. The internal standard N-pentadecane was used in calculating the quantity of other compounds. Thermo Scientific TRACE 1300 Gas Chromatograph and TSQ 8000 Evo Triple Quadrupole Mass Spectrometer (Thermo Fisher Scientific Inc., Waltham, MA, USA) were used to detect and analyse for GC-MS, with TG-5 MS column (30 m × 0.25 mm, 0.25 μm film thickness; Thermo Fisher Scientific Inc., Waltham, MA, USA) as the chromatographic separation column. Retention in dice (RI) was calculated by using the C7–C30 n-alkanes standard. The compounds were identified by comparison with mass spectra reference library NIST MS 2010, and using RI match, the data were processed by Thermo scientific Xcalibur data analysis system version 4.2.47 [48].

### 4.8. Gene Expression Quantification

The relative expression levels of selected genes were determined using reverse transcription quantitative PCR (RT-qPCR) to verify the reliability of RNA-Seq data. Total RNA was isolated as described above, and one microgram of total RNA was reverse-transcribed using the cDNA Synthesis Kit (Takara Biotechnology, Dalia, Liaoning, China). RT-qPCR was performed using Hieff UNICON^®^ Universal Blue RT-qPCR SYBR Green Master Mix (Yeasen Biotechnology Shanghai, China) according to the manufacturer’s protocol and run in a LightCycler 480 II Real-Time System R (Roche Diagnostics, Basel, Switzerland) [49]. The expression level of genes was calculated by the 2^−ΔΔCt^ method with normalisation based on the reference gene *OsEF1-alpha* [50]. All genes were repeated three times, with three samples per replicate. The primers used in this study are shown in Table S11.

## 5. Conclusions

This study used the WTV (widely targeted method for plant volatilome study) method combined with the transcriptome to explore VOCs in rice under SSB infestation. Next, we examined and discussed the functions of the selected VOCs and related genes associated with plant defence. We also explored the influence of VOCs on SSB behaviour and feeding performance. These findings provide an understanding of the candidate VOCs involved in the rice response, especially in chemical defence against herbivores, to improve rice resistance.

## Data Availability

The data that support the findings of this study are available from the corresponding author upon reasonable request.

## Supplementary Materials

The following supporting information can be downloaded at: https://www.mdpi.com/article/10.3390/ijms25168827/s1.
