# Supplementary material for: Infestation of Rice Striped Stem Borer (Chilo suppressalis) Larvae Induces Emission of Volatile Organic Compounds in Rice and Repels Female Adult Oviposition
Source: Int J Mol Sci. 2024 Aug 13;25(16):8827. doi: 10.3390/ijms25168827 (PMC11354779; doi:10.3390/ijms25168827)
Supplement: Supplementary file 1 [file ijms-25-08827-s001.zip › Abbreviation list.docx]

| Abbreviation | Full Name |
| --- | --- |
| SSB | strip stem borer |
| HIPVs | herbivore-induced plant volatiles |
| OSs | oral secretions |
| VOC | volatile organic compound |
| WTV | widely targeted method for plant volatilome studies |
| HS-SPME | Headspace solid-phase microextraction |
| GC-MS | chromatography-mass spectrometry |
| PCA | principal component analysis |
| DEGs | differentially expressed genes |
| HCA | hierarchical cluster analysis |
| GO | gene ontology |
| KEGG | kyoto encyclopedia of genes and genomes |
| OPLS-DA | OSC-partial least squares-discriminant analysis |
| FPKM | fragments per kilobase of transcript per million fragments mapped |
| VIP | Variable Importance in Projection |
