# Supplementary material for: Infestation of Rice Striped Stem Borer (Chilo suppressalis) Larvae Induces Emission of Volatile Organic Compounds in Rice and Repels Female Adult Oviposition
Source: Int J Mol Sci. 2024 Aug 13;25(16):8827. doi: 10.3390/ijms25168827 (PMC11354779; doi:10.3390/ijms25168827)
Supplement: Supplementary file 1 [file ijms-25-08827-s001.zip › Figure S10.docx]

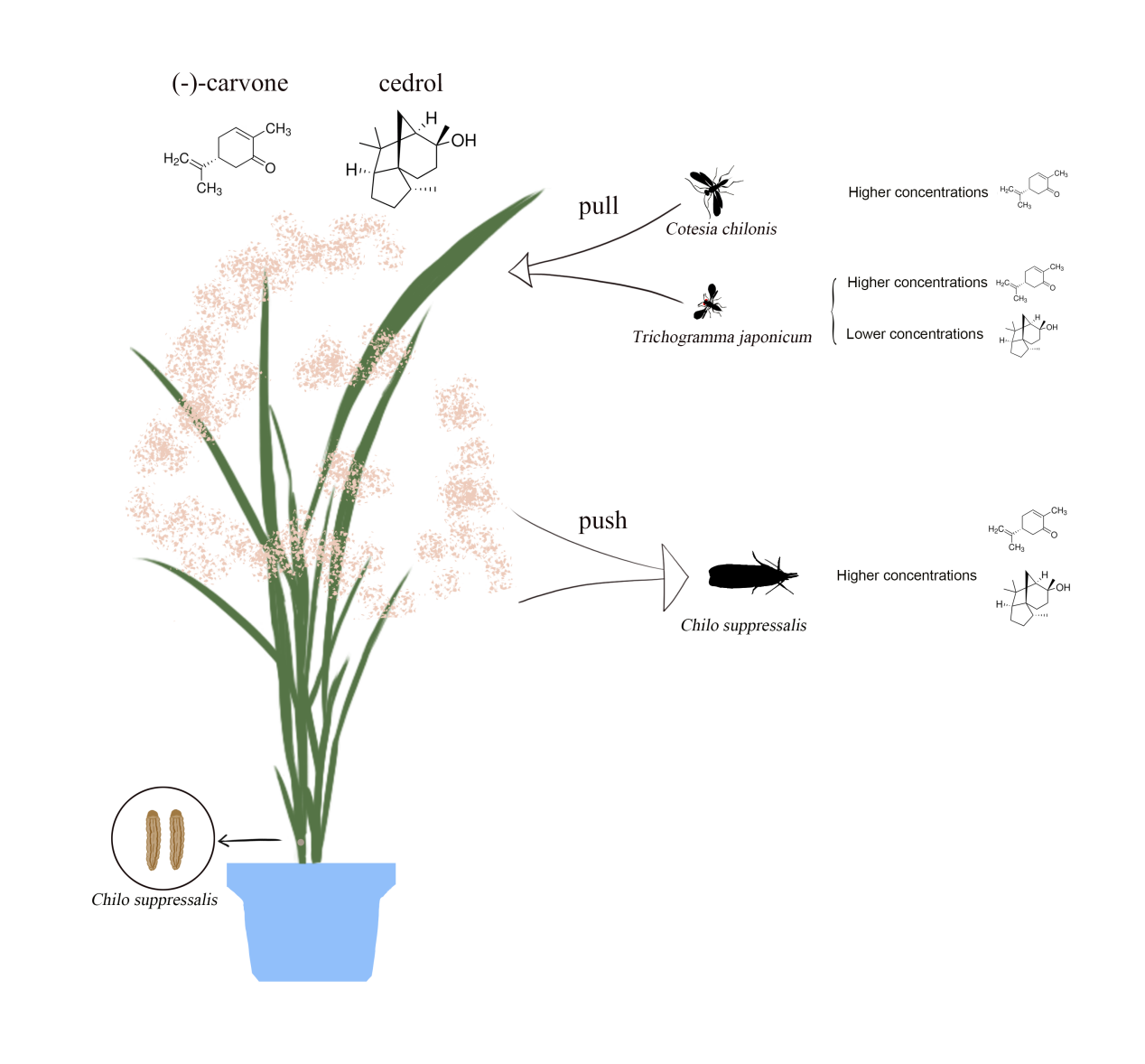


Figure S10. A scheme summarising the interactions between rice plants, SSB, and natural enemies throughout the VOCs
