# Supplementary material for: Infestation of Rice Striped Stem Borer (Chilo suppressalis) Larvae Induces Emission of Volatile Organic Compounds in Rice and Repels Female Adult Oviposition
Source: Int J Mol Sci. 2024 Aug 13;25(16):8827. doi: 10.3390/ijms25168827 (PMC11354779; doi:10.3390/ijms25168827)
Supplement: Supplementary file 1 [file ijms-25-08827-s001.zip › Figure S8.docx]

A


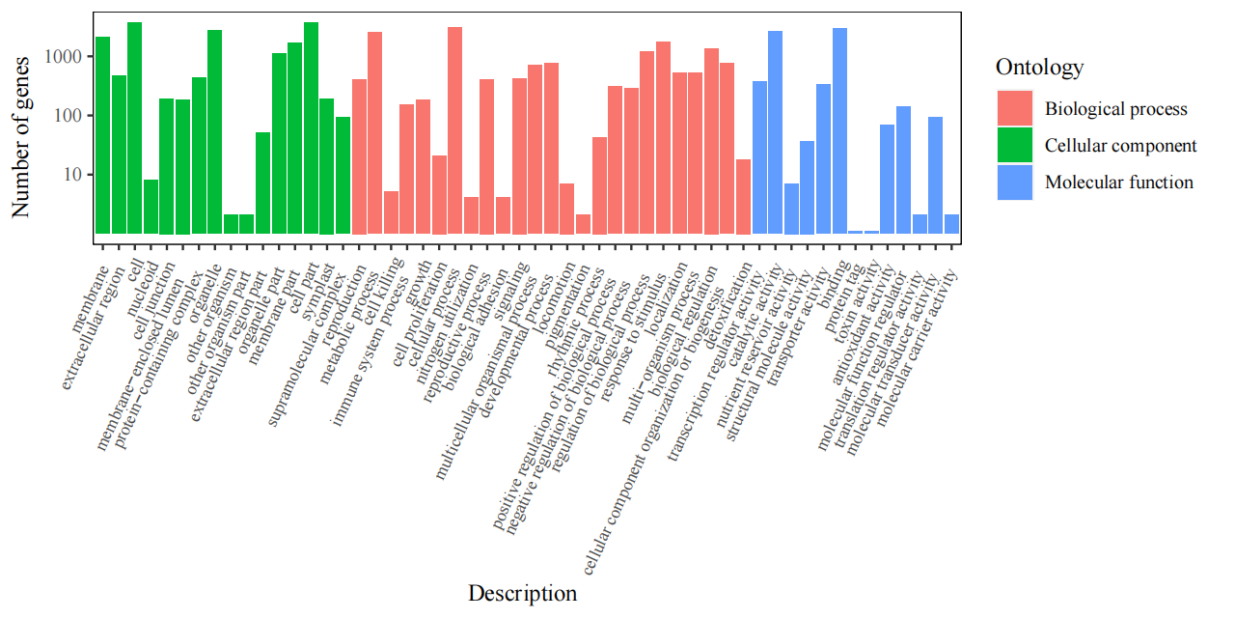


B


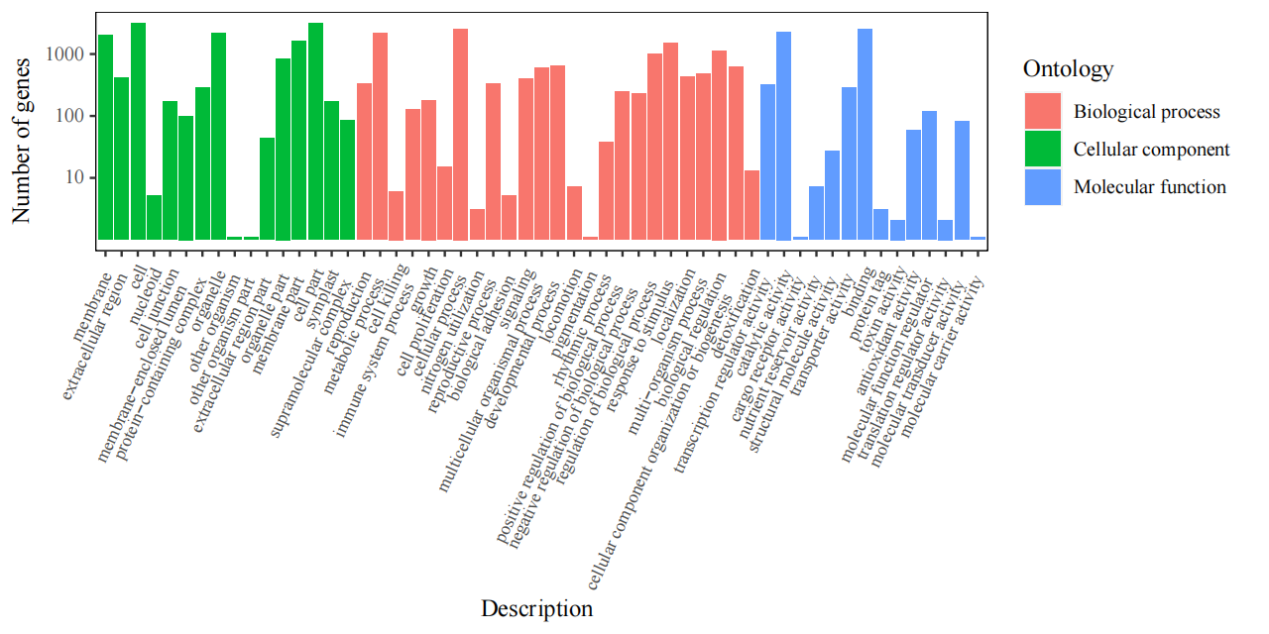
Figure S8. Statistics of Gene Ontology (GO) enrichment of differentially expressed genes at the 24 h and 48 h time-point compared with the control group. *p*≤0.05 and absolute value of Log_2_FC (fold-change) ≥1.0 are used as the threshold for significant difference expression.
