# Supplementary material for: Infestation of Rice Striped Stem Borer (Chilo suppressalis) Larvae Induces Emission of Volatile Organic Compounds in Rice and Repels Female Adult Oviposition
Source: Int J Mol Sci. 2024 Aug 13;25(16):8827. doi: 10.3390/ijms25168827 (PMC11354779; doi:10.3390/ijms25168827)
Supplement: Supplementary file 1 [file ijms-25-08827-s001.zip › Figure S9.docx]

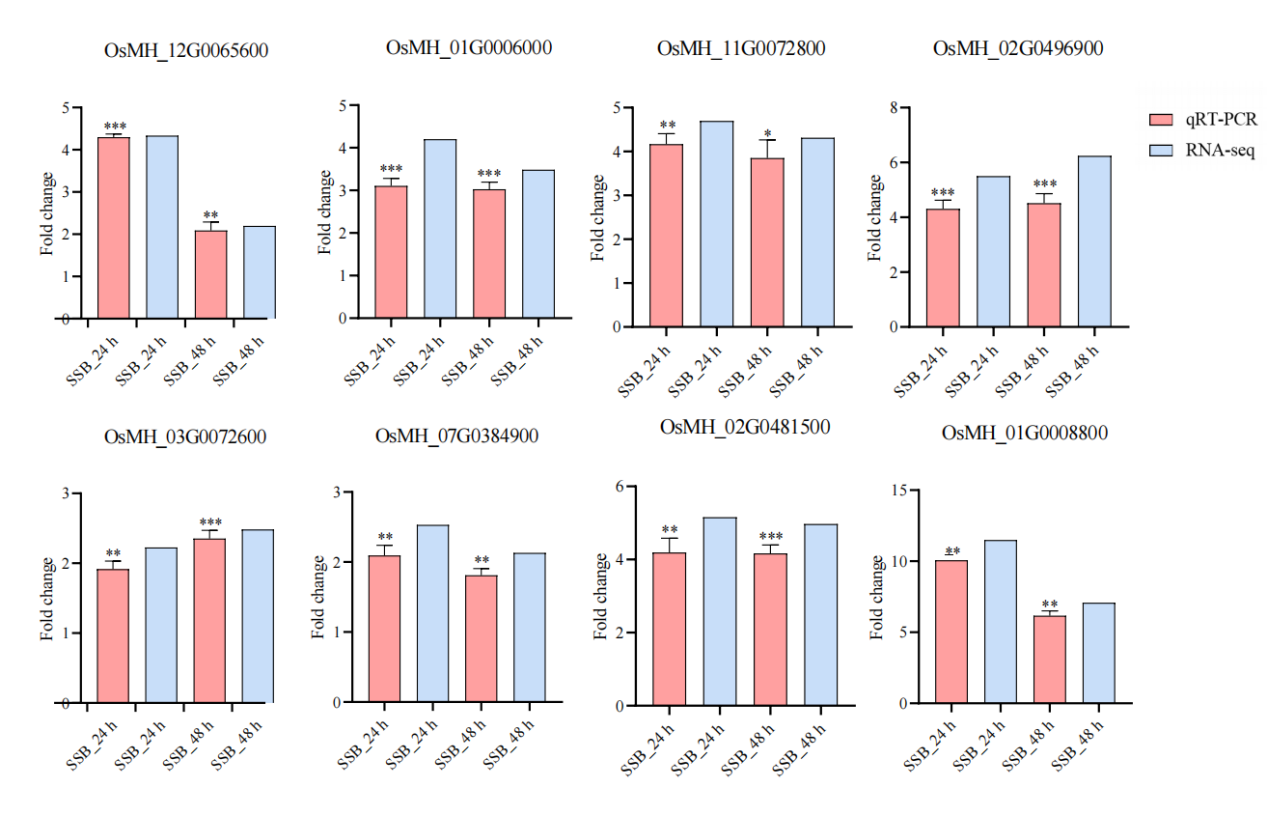


Figure S8. Validation of the gene expression profiles obtained by RNA-seq using quantitative polymerase chain reaction. Fold-changes in transcript abundance obtained by both qRT-PCR and RNA-seq were presented on the same graph for eight genes. The red column represent the qRT-PCR results and blue column represent the RNA-seq results. * *p* < 0.05, ** *p* < 0.01, *** *p* < 0.001.
