# Supplementary figures and images for: Infestation of Rice Striped Stem Borer (Chilo suppressalis) Larvae Induces Emission of Volatile Organic Compounds in Rice and Repels Female Adult Oviposition
Source: Int J Mol Sci. 2024 Aug 13;25(16):8827. doi: 10.3390/ijms25168827 (PMC11354779; doi:10.3390/ijms25168827)

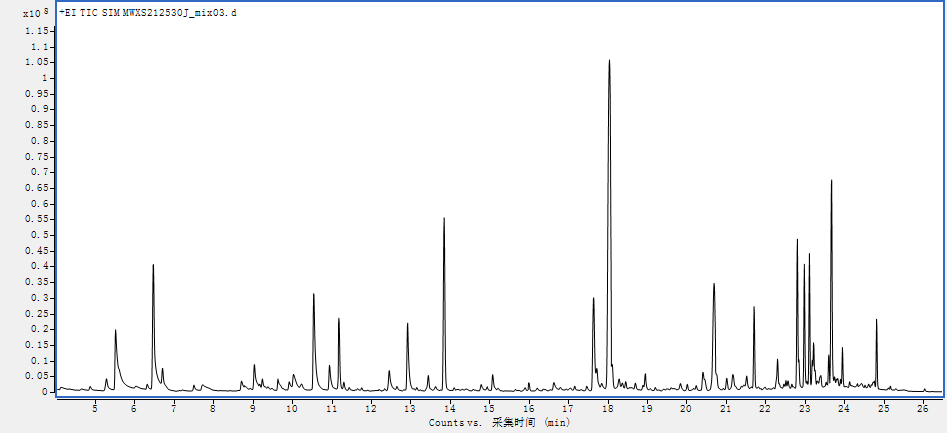


Figure S1. Sample mass spectrometry analysis of total ion chromatogram

Supplement: Supplementary file 1 [file ijms-25-08827-s001.zip › Figure S1.docx]

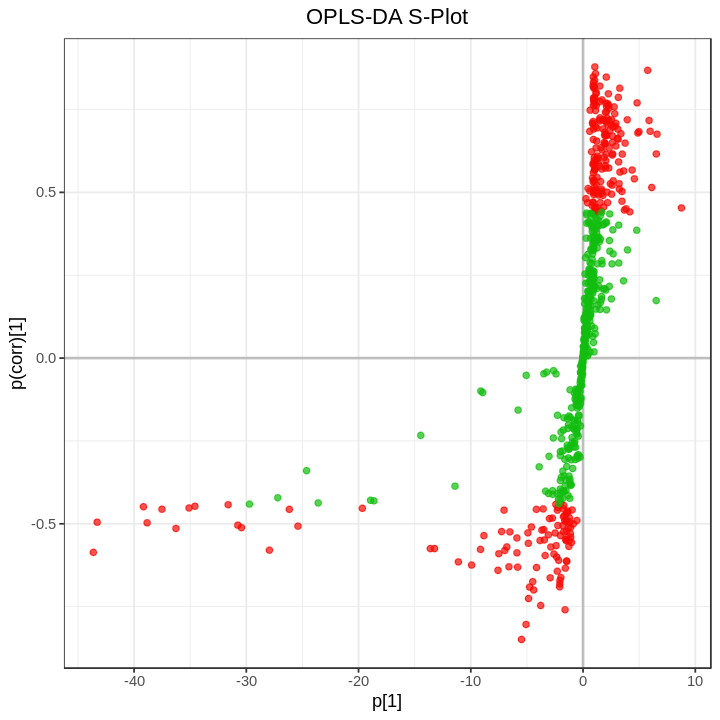


A


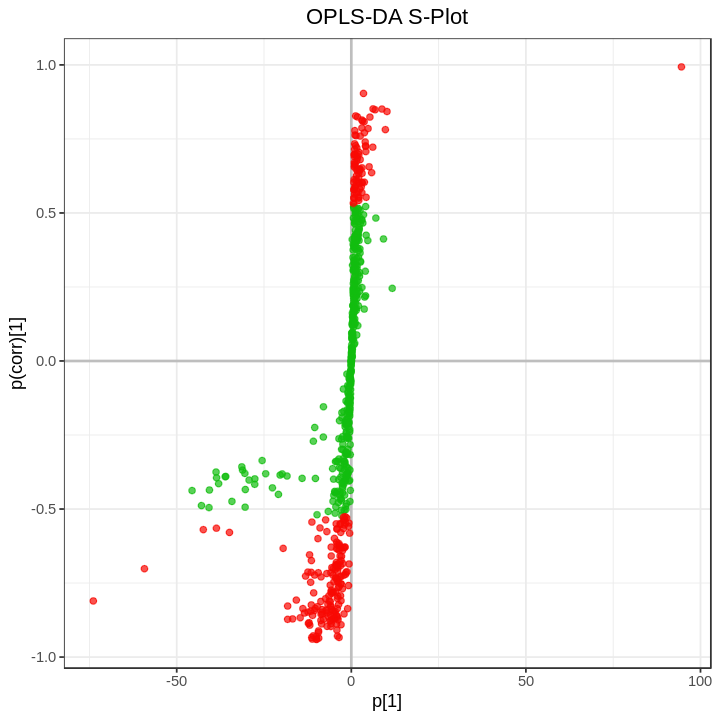


B

Figure S3. OPLS-DA S-plot A: SSB_24 h vs. Control. B:SSB_48 h vs. Control

Supplement: Supplementary file 1 [file ijms-25-08827-s001.zip › Figure S3.docx]

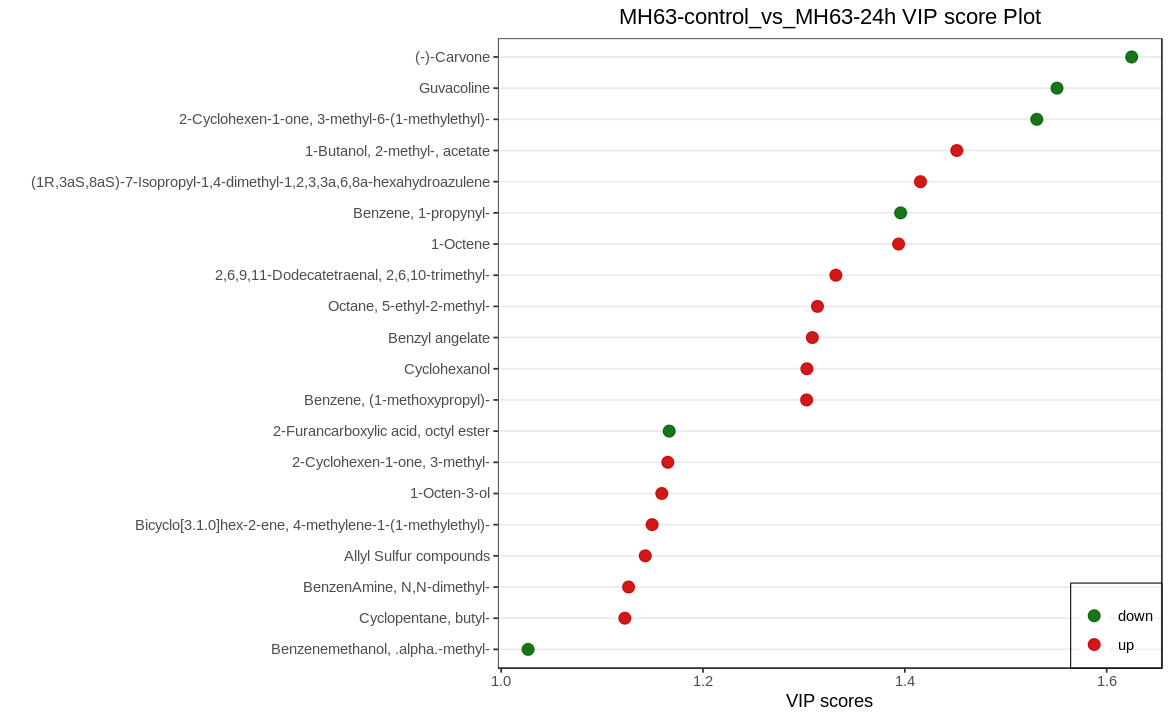


A


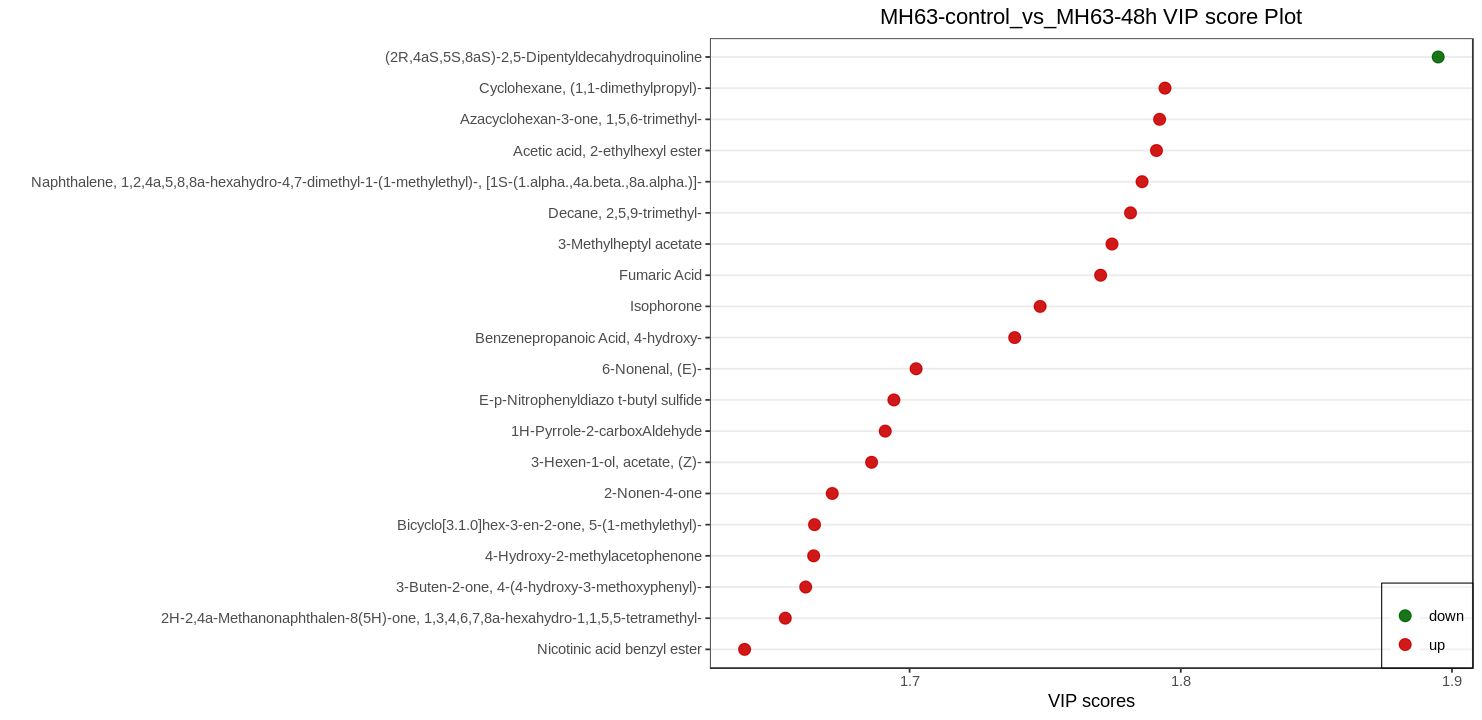


B

Figure S4. VIP Value chart of differential metabolites. A: SSB_24 h vs. Control. B: SSB_48 h vs. Control.

Supplement: Supplementary file 1 [file ijms-25-08827-s001.zip › Figure S4.docx]

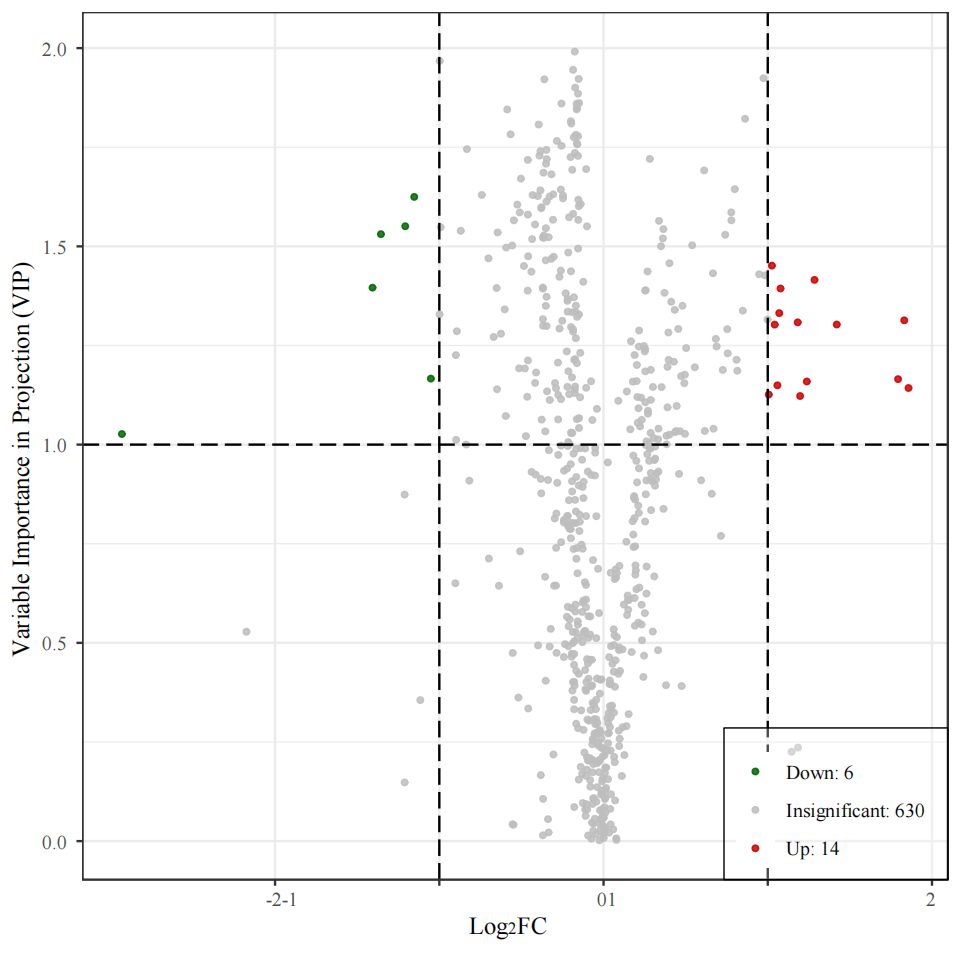


A


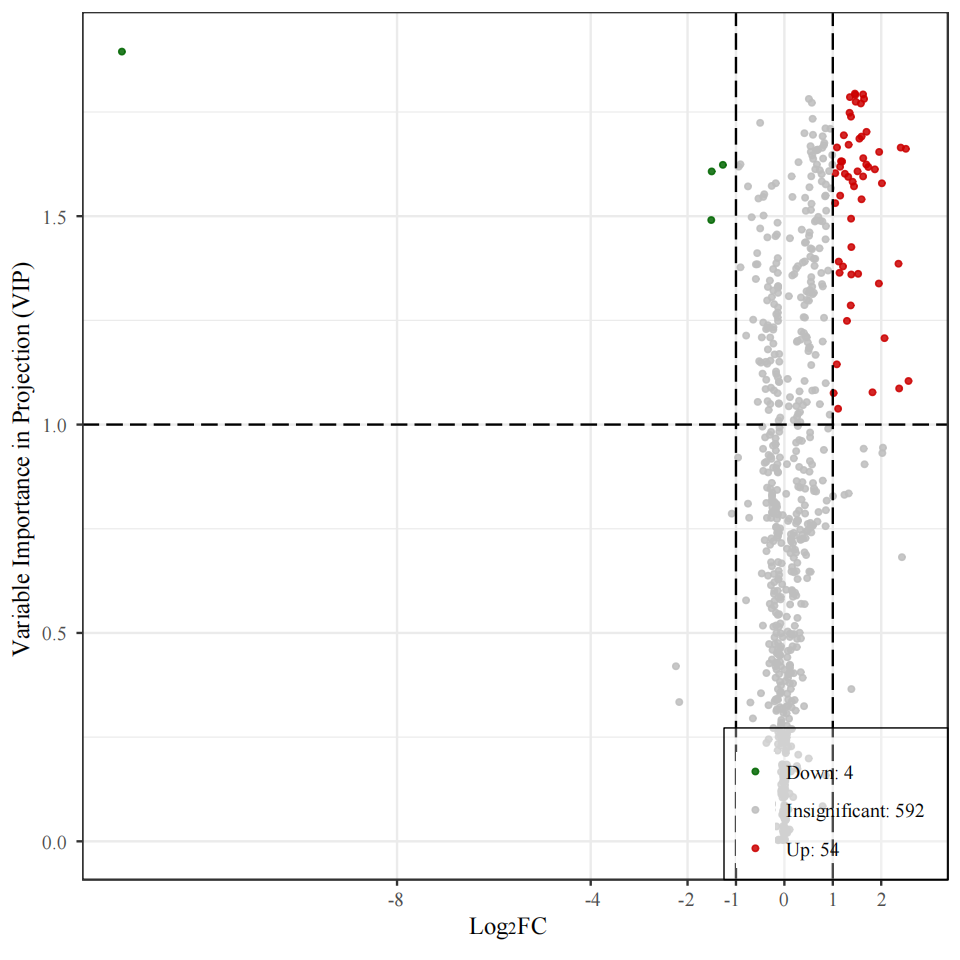


B

Figure S5. Volcano Plot.A: SSB_24 h vs. Control. B: SSB_48 h vs. Control.

Supplement: Supplementary file 1 [file ijms-25-08827-s001.zip › Figure S5.docx]

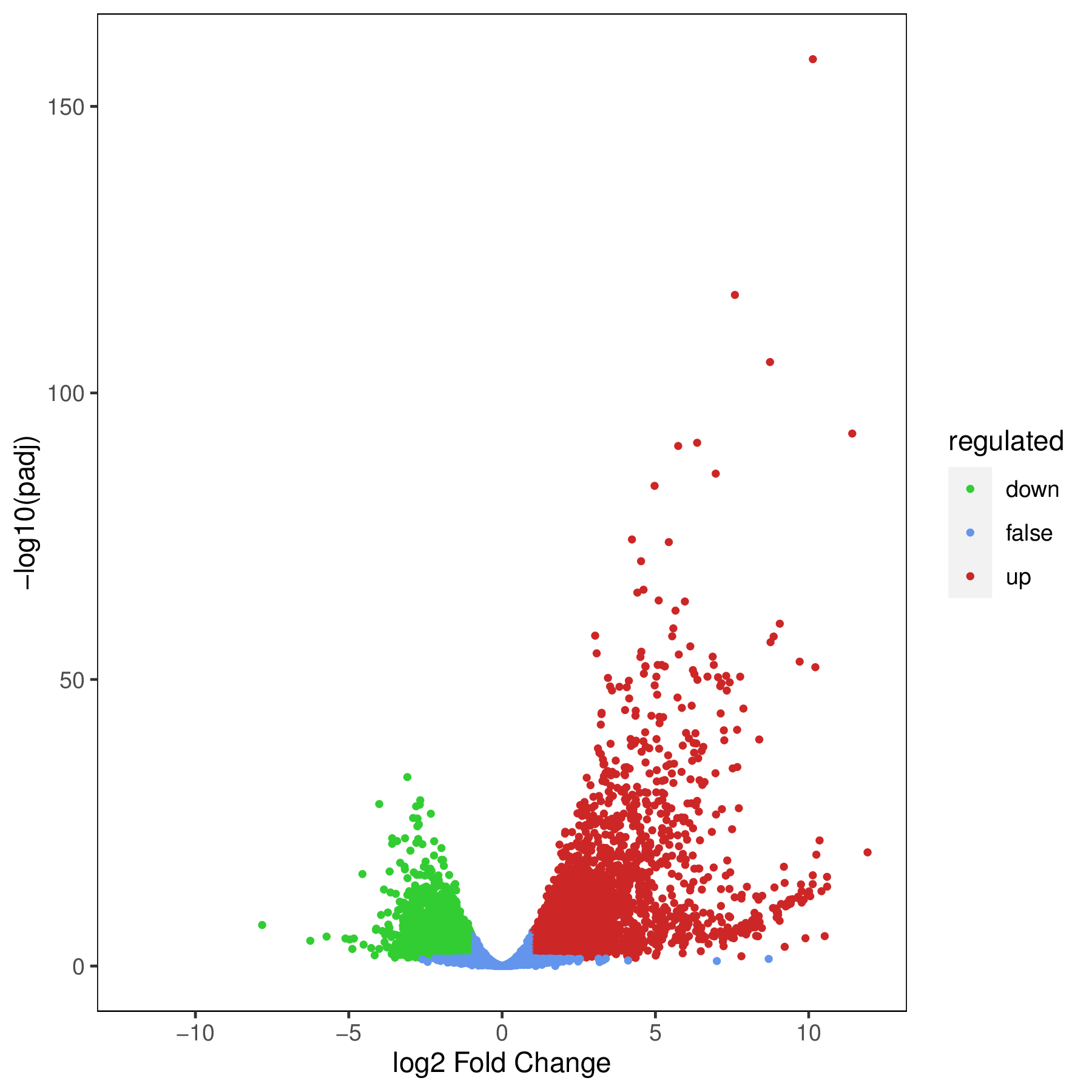


A


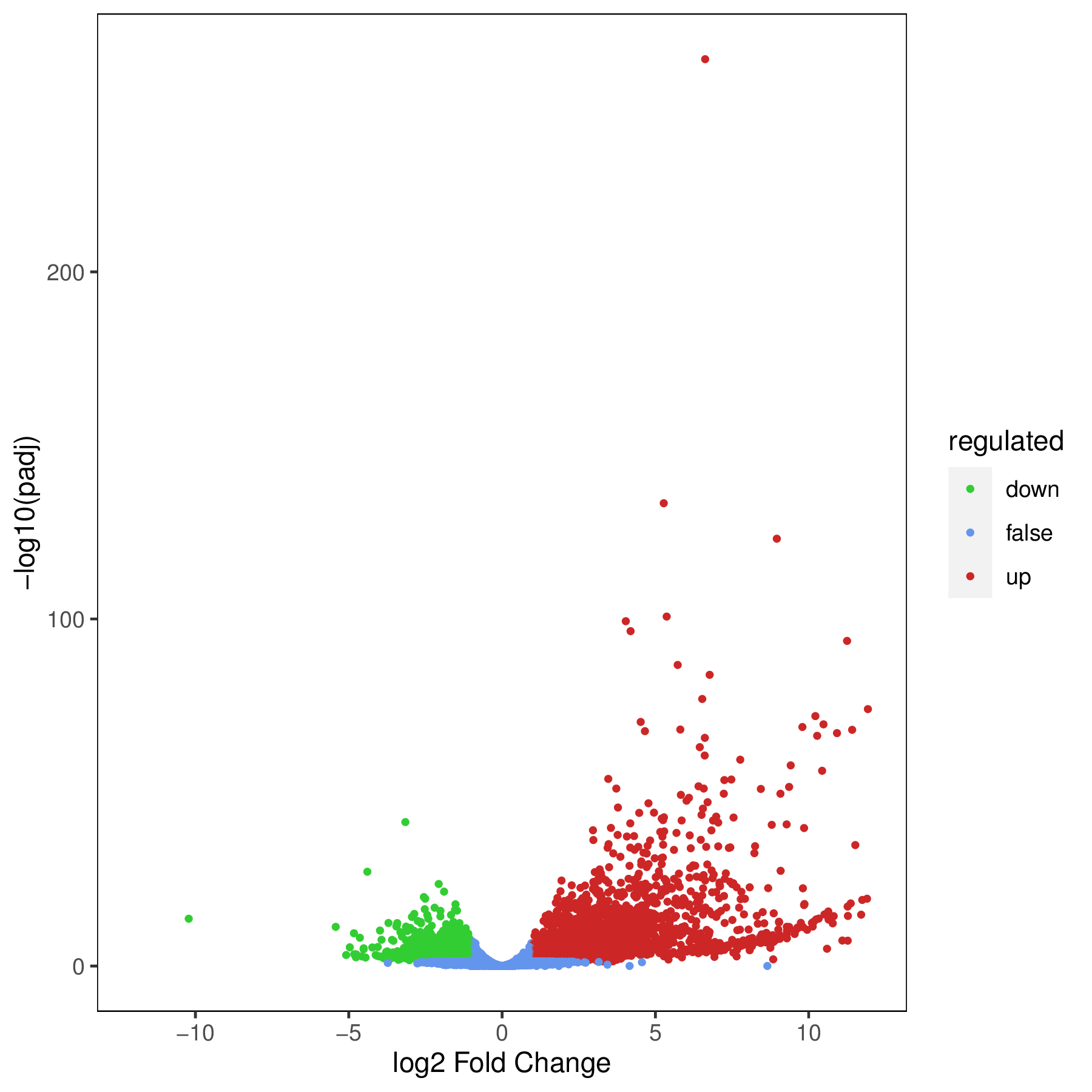


B

Figure S7. Differential gene volcano plot.A: SSB_24 h vs. Control. B: SSB_48 h vs. Control.

Supplement: Supplementary file 1 [file ijms-25-08827-s001.zip › Figure S7.docx]
